# Silicon in prevention of atherosclerosis and other age-related diseases

**DOI:** 10.3389/fcvm.2024.1370536

**Published:** 2024-03-01

**Authors:** Łukasz Dudek, Wacław Kochman, Ewelina Dziedzic

**Affiliations:** ^1^ Department of Cardiology, Bielanski Hospital, Warsaw, Poland; ^2^ Cardiovascular Clinic, Centre of Postgraduate Medical Education, Warsaw, Poland

**Keywords:** silicon, orthosilicic acid, atherosclerosis, glycosaminoglycan, inflammation

## Abstract

Researchers' interest in silicon as an element important for the functioning of the animal and human body began in the 1970s. Soluble compounds of silicon bioavailable from water and food seem to have important meaning for life processes occurring in the body. So far, researchers have focused on the significance of silicon for the development of bones and connective tissue, and its role in preventing neurodegenerative diseases and atherosclerosis. Despite numerous studies, the role of silicon as an active element in the human body is poorly understood. Since the involvement of lipid oxidation and inflammatory processes in the pathogenesis of atherosclerosis is well known, this article summarizes and discusses the current research and scientific observations regarding silicon, primarily in terms of its beneficial influence on redox and anti-inflammatory reactions and the lipid profile. The association of silicon with the stabilization of the structure of glycosaminoglycans and their protein complexes may also support the anti-atherosclerotic effect. The authors attempted to collect and present existing publications that could confirm the beneficial role of dietary silicon in the prevention of age-related diseases and explain the potential mechanisms of its action.

## 1 Introduction

Atherosclerosis is a chronic inflammatory disease of the arterial wall, the complications of which (heart attack and cerebral stroke) are the most common cause of death in developed countries (1). Despite the relatively well-understood pathomechanism and the availability of drugs that slow down the formation of atherosclerotic plaques, there are still many questions regarding its development and new agents with anti-atherosclerotic potential are constantly being searched for. For several decades, the role of silicon in preventing the development of some chronic diseases has been discussed in scientific literature, and reports about the possible role of the element in preventing atherosclerosis date back to the 1970s (2, 3). Researchers indicate the possible role of silicon for the proper functioning of the cardiovascular, skeletal and central nervous systems (4, 5). Taking into account the prevalence of the element in the environment, we reviewed existing scientific work with the aim of answering the question whether silicon as a molecular component can modify/influence the course of atherosclerosis and other age-related diseases.

## 2 Sources of silicon for the human body and its bioavailability

The main source of silicon for the human body is diet based on natural products containing the bioavailable forms of Si. Although the element is widespread in nature as a component of earth's minerals, rocks, sands, clays, dust and also widely used in industry (inter alia cosmetics, medical implants, medical devices, electronics, computer devices, metallurgy), environmental exposure to humans is quite limited due to the difficult absorption of present in nature or industrially used chemical forms of silicon. However, forms of the element commonly used in the food industry, such as amorphous silica (SiO_2_), silicates or polymethylsiloxane occurring as food additives, clarifying agents in drinks, ingredients of medicines and vitamins, can be significant source of the silicon for the human body (6, 7). Silicon is also present in the human diet in the form of highly soluble forms derived from water and natural products.

To date, no minimum daily requirement for silicon has been established. However, the recommended daily intake of the element was estimated at 10–25 mg/day taking into account the daily urinary excretion of silicon (8).

Silicon the most often occurs as poorly soluble mineral silica (SiO_2_) in a polymerized form (9). When exposed to water, silicates are formed, which release orthosilicic acid Si(OH)4' to a concentration of 1–15 mg/L SiO_2_. In this form silicon is bioavailable and easily absorbed in the digestive tract. The bioavailability of silicon varies depending on the food: orthosilicic acid and water-soluble silicates from beverages are easily absorbed, while phytolytic silica, present in solid plant foods, is less digestible (10).

The high silicon excretion after beer consumption indicates that it is a particularly important source of easily absorbable silicon (11). The beer is rich in bioavailable silicon from hot mashing techniques that extract orthosilicic acid from the phytolitic silica of barley. Other beverages are also indicated as sources of bioavailable silicon, especially tap water, mineral and spring waters (7).

Silicon levels are usually higher in plant-based foods than in animal-based foods. Foods with the highest silicon content are cereals, especially oats, barley and some rice fractions (12). Cereal grains, along with beverages (especially beer) and some vegetables and fruits, contribute the most to dietary silicon intake (8).

## 3 The importance of silicon in cardiovascular diseases

Silicon is an essential trace element that occurs in the human body in small amounts. In serum, the element occurs almost entirely as orthosilicic acid. Concentrations of Si in plasma in fasting state are 2–10 µM, growing to 20–30 uM after meals, and approximately 700 μmol/day is normally excreted in urine (13). Serum silicon levels in adults shows statistically significant dependency on age and sex. According to Bisse et al. (14) the decrease of serum silicon with age is clearly visible over 74 years, especially in women.

Some reports have observed also age-related lowering the silicon content in the hair of healthy people (15).

### 3.1 The effect of silicon on the structure of arteries and glycosaminoglycans

Connective tissues, including the aorta and trachea are extremely rich in silicon, as has been shown by studies on several animal species (16). The high content of silicon in connective tissue is mainly due to its participation in the formation of glycosaminoglycans, as well as the presence of the element as an integral element of glycosaminoglycans and their protein complexes. Proteoglycans are components of the extracellular amorphous ground substance surrounding cells, collagen and elastic fibers, forming the structure of this tissue. The structural role of silicon in the extracellular matrix, which involves cross-linking proteoglycans and protein, increases the strength and reduces the permeability of the matrix (17), thus strengthening the arterial walls. Interaction of proteoglycans with LDL leading to subendothelial retention of LDLs has been proposed to be a key process in the pathogenesis of atherosclerosis (18, 19). It cannot be ruled out that strengthening the proteoglycans structure can limit the availability of the negative sulfate groups of glycosaminoglycans for binding sites of apoB protein in LDL. Protection of the proteoglycan structure by silicon may contribute to the prevention of atherosclerosis not only by improvement the function of the arterial wall, but also by the action of the proteoglycans themselves. The results of current scientific research indicate that certain types of heteroglycans have a beneficial effect in the prevention of metabolic syndrome. Administration of medium and high doses of heteropolysaccharides to rats with metabolic syndrome (MetS) significantly reduced both systolic and diastolic blood pressure (*p* < 0.05), improved cardiac ejection fraction (*p* < 0.05) and contractility (*p* < 0.05) compared to untreated MetS rats (*p* < 0.05). Supplemented MetS rats also demonstrated amelioration of the lipid status with statistically significant decrease in triglycerides, LDL cholesterol, total cholesterol and increase in HDL cholesterol compared to untreated MetS group. An improvement in glucose homeostasis in the OGTT test and decrease in insulin levels (*p* < 0.05) were also observed (20).

Research results have confirmed that aging causes a marked reduction in the level of this element also in the normal human aorta (2), which may have an adverse effect on the structure of arterial blood vessels in older people.

### 3.2 Silicon and the development of atherosclerosis

Loeper et al. (2), found that the level of silicon in the wall of the human aorta decreases with the development of atherosclerosis. The silicon content in the aorta with moderate atherosclerosis (1–2 atherosclerotic plaques present) was approximately 58%, and in the aorta with severe atherosclerosis (numerous atherosclerotic plaques and massive lipid infiltrates present) was approximately 35% of the content in the aorta without atherosclerosis. The decrease in silicon content paralleled lipid infiltration, changes in elastic fibers, and changes in the ground substance. A very significant difference was found between group 0 (aorta without atherosclerosis, 0 atherosclerotic plaque) and group + (aorta with moderate atherosclerosis, 1–2 atherosclerotic plaques), as well as between group + and group ++ (aorta with severe atherosclerosis, numerous atherosclerotic plaques and massive lipid infiltrates), with *p* < 0.001. Similar results were obtained in study of rabbit aortas (21) (Table 1).

**Table 1:** Summary of works on the assessment of silicon in atherosclerosis based on pathology.

Authors	Materials	Groups	Results		
			Si concentration in the aorta (Silicon in µg/100 mg nitrogen)	*p*	Others
Loeper et al. (2)	human aorta	Group 0 aorta without atherosclerosis, 0 atherosclerotic plaque	180 µg ± 21	<0.001 (group 0 vs. group +) (group + vs. group ++)	–
		Group (+) aorta with moderate atherosclerosis, 1–2 atherosclerotic plaques	105 µg ± 12		
		Group (++) aorta with severe atherosclerosis, numerous atherosclerotic plaques and massive lipid infiltrates	63 µg ± 8		
Loeper et al. (21)	rabbit aorta	Group 1 normal aorta from rabbits on standard diet	490 µg ± 40	<0.05 (group 1 vs. group 2)	Among all rabbits fed a high-cholesterol diet (Group 2 + Group 3), atherosclerotic lesions occurred in 30% of rabbits treated with silicon, while in 88% of untreated rabbits.
		Group 2 aorta with experimental atherosclerosis induced by high cholesterol diet	398 µg ± 66		
		Group 3 aorta with experimental atherosclerosis induced by high cholesterol diet with addition of silicon i.v. (monomethyltrisilanol 10 mg every other day)	470 µg ± 53		
Gangapatnam et al. (22)	rats	Group I normal control rats	–	–	Histopathological studies were estimated. Silicon at 20 mg/ml concentration showed the regression of atherosclerosis in high fat diet induced rats compared to 10 mg/ml concentration.
		Group II high fat diet rats			
		Group III statins as positive control			
		Group IV high fat diet + silicon treatment with 10 mg/ml			
		Group V high fat diet + silicon treatment with 20 mg/ml concentration.			

Human study assessed the silicon content also in the hair of patients with atherosclerosis (15). Silicon levels were compared in the same age groups of healthy patients and those suffering from atherosclerosis. Statistically, no major differences were found, but the mean silicon hair content in patients with atherosclerosis was much lower and ranged from 14.0 ± 6.7 to 7.9 ± 4.9 µg/g, depending on the age range. A wide range of silicon values was observed in each age group, even in the healthy military students' group living for many years in the same conditions and using the same diet. However, among patients with atherosclerosis, a group was found with very low levels of silicon in the hair, below 10 µg/g. Due to the wide range of concentrations in the samples, it is risky to consider this parameter as a marker of the disease, but a very low silicon hair content below 10 µg/g may be a clear indicator of the disease.

The potential contribution of silicon deficiency to atherosclerosis was also indicated by other researchers (3, 23, 24). The authors suggest that poorly soluble forms of polymeric silicic acid or silica may reduce the development of atherosclerosis by binding bile acids in the gastrointestinal tract, which would improve the elimination of end products of cholesterol metabolism; the binding of cholesterol itself may also play a role. The proposed concept may be confirmed by studies that have shown that the addition of silicic acid to drinking water increases the excretion of tritium-labeled cholesterol and its transformation products in the feces and reduces the absorption of labeled cholesterol in the liver, spleen and kidneys (3). The results of other study suggest that silicon among other metal ions (calcium, magnesium, lithium, strontium) may protect against cardiovascular mortality; possibly, by competing with sodium and potassium for transport in the gastrointestinal tract (23).

Epidemiological studies have revealed many genetic and environmental risk factors for atherosclerosis (25). Genetic factors include: dyslipidemia, increased LDL/VLDL levels, decreased HDL, increased blood pressure, increased homocysteine levels, diabetes and obesity, systemic inflammation (elevated CRP), metabolic syndrome with insulin resistance, male gender. Environmental factors include primarily a high-fat diet, smoking, low levels of antioxidants, low physical activity, and some infectious factors.

### 3.3 Silicon and the lipid profile

The effect on lipids is considered as one of the mechanisms of the anti-atherogenic effect of silicon. In studies on rabbits (21), it was found that 88% of rabbits on an atherogenic diet developed atherosclerosis, while on an atherogenic diet with intravenous silicon addition, only 30% of rabbits showed atherosclerotic plaques. In the group of animals receiving silicon, the increase in the plasma concentration of mono- and polyunsaturated fatty acids was lower compared to the group not treated with silicon. It seems that silicon may have a beneficial effect on atherosclerosis by reducing mono- and polyunsaturated fatty acids in lipids, which in the peroxidation process release toxic peroxides that cause damage to arteries. A study by Najda et al. (26) showed that the concentration of HDL-cholesterol and HDL-phospholipids increased and the concentration of triglycerides and LDL-cholesterol decreased in the blood serum of rats supplemented with an aqueous silica solution. However, there was no effect of silicon supplementation on the plasma concentration of total cholesterol or phospholipids. However, Garcimartin et al. (27) showed in animal studies that in the group receiving the hypercholesterolemic + silicon diet, the levels of cholesterol (C), phospholipids (PL) and triglycerides (TG) were significantly lower compared to the group receiving only the hypercholesterolemic diet. Cholesterol supplementation increased C and PL in very low-density lipoprotein (VLDL) and intermediate-density lipoprotein + low-density lipoprotein (IDL + LDL). In the group on the hypercholesterolemic + silicon diet a reduction in the total VLDL mass (*p* < 0.001) to the value of the control group receiving a normal diet, a decrease in the atherogenic index (AI) and a decrease in the share of TG in all lipoproteins in relation to the other two groups were observed.

### 3.4 Silicon and blood pressure

Arterial hypertension is a known risk factor for the development of atherosclerosis. In a study conducted by Maehira et al. (28) using soluble silica and coral sand as a natural material containing silicon, the effect of silicon on hypertension in spontaneously hypertensive rats was assessed. In rats fed with soluble silica (at a dose of 50 mg/kg body weight) or coral sand for 8 weeks, systolic blood pressure was significantly reduced respectively by 18 mmHg or 16 mmHg compared to control group. Moreover, providing both dietary soluble silica and coral sand inhibited the expression of angiotensinogen and aortic growth factor genes associated with vascular remodeling. Soluble silica also stimulated the expression of peroxisome proliferator-activated receptor γ, which has anti-inflammatory and antihypertensive effects on blood vessel cells.

### 3.5 Possible effect of silicon on the functions of endothelial cells

Atherosclerosis is a chronic inflammatory process taking place in the intima of large and medium-sized arteries. Some authors suggest that dietary silicon may influence endothelial cells and maintain the proper inner lining of arteries. This is related to abundant evidence for the physiologically important role of heparan sulfate-containing proteoglycans (HSPG) produced by vascular endothelial cells in inhibiting the morphological transformation, migration and proliferation of vascular smooth muscle cells, i.e., processes that are crucial for the intimal hyperplasia observed in atherosclerosis and restenosis (29). It has been suggested that oral administration of glucosamine may stimulate the production of heparan sulfate-containing proteoglycans and thus exert antiatherosclerotic effects. Similarly, the authors suggest that the intake of bioavailable silicon may also increase the production of heparan sulfate-containing proteoglycans by endothelium and that this phenomenon, at least partially, underlies the beneficial effect of silicon administration on the inhibition of intimal hyperplasia in cholesterol-fed rabbits and may also explain the lower cardiovascular risk associated with increased silicon intake in epidemiological studies. To evaluate this hypothesis it is suggested to study the effect of soluble silicates on production of HSPG by cultured cells of vascular endothelium. But also reports with contrary results showing potentially adverse effects of Si for function of endothelial cells and decreased endothelial NO synthase expression are present in scientific literature (30). The experimental study has shown that contractility induced by norepinephrine and dilation response to acetylcholine were significantly higher in the aortic rings of Si-treated rats compared to controls. The study demonstrated that Si modified the characteristics of endothelial relaxants and attenuated smooth muscle cell responsiveness to NO caused by decrease of production of basal NO generation (31).

### 3.6 Silicon and inflammatory processes/the function of the immune system

The formation of atherosclerotic plaque is a complex process of oxidative, inflammatory and immunological origin, which involves both activated macrophages and T lymphocytes, as well as the production of numerous cytokines and growth factors (32). Results of studies on silicon-deprived animals suggest the element's involvement in immune and inflammatory reactions (33, 34). The role of silicon in enhancing the anty-inflammatory response and modifying the immune response has so far been observed in a study on animals with collagen-induced arthritis (33). During inflammation, the number of lymphocytes decreased and the number of neutrophils increased significantly in rats whose diet was supplemented with silicon, compared to rats on the basic diet. The importance of these changes in the context of the pathogenesis of atherosclerosis is ambiguous, but the lack of significant increase in monocyte levels in the group with silicone supplementation seems to be beneficial. There was also increased release of prostaglandin E2 (PGE2) in the tibia and higher plasma osteopontin levels in rats supplemented with silicon, suggesting a weaker anti-inflammatory response in rats fed a diet without added silicon.

No differences were found in cellular parameters and levels of pro-inflammatory cytokines in the initial acute phase (2 h) of the inflammatory process induced by lipopolysaccharide endotoxin in rats deprived of silicon compared to those supplemented. However, it was observed that after exposure to endotoxin, rats deprived of silicon accumulated more silicon in the liver and bones than rats receiving silicon supplements, which may suggest its involvement in the response to the chronic inflammatory process. Moreover, in the control group of rats deprived of silicon, there was an increase in the number of monocytes (35), cells of key importance in the process of initiating atherosclerosis.

In mouse induced macrophage cells, the inhibitory effects of silicon on nitric oxygen generation and on IL-6 secretion were observed at addition respectively of 100 µM and of 50 µM of Si in sodium metasilicate form, significantly exceeding the concentration of Si found in human blood. But significant decrease in mRNA expression of tumor necrosis factor TNF-α and inducible nitric oxide synthase level by 1 µM, 5 µM and 10 µM of Si in sodium metasilicate form and also suppression of cyclooxygenase-2 mRNA expression by 1 µM, 5 µM and 25 µM of Si in the same chemical form suggest ability of silicon to regulate the inflammatory process (36). Similarly to neurodegenerative diseases, demonstrated by Garcimartin et al. (37) in *in vitro* studies, a reduction in the level of tumor necrosis factor TNF-α in response to low concentrations of silicon and found by Gonzalez-Munoz et al. (38) in a study on mice, normalization of TNF-α mRNA expression after the use of silicic acid may be important in atherosclerosis.

In 2015, Vide et al. (39) studied the effect of dietary silicon-enriched spirulina (SES) on atherosclerosis. Spirulina are cyanobacteria that live in highly saline tropical and subtropical waters. These microalgae are one of the richest plant sources of proteins (60%–70%), lipids (7%), carbohydrates (20%) and good source of vitamins and minerals such as calcium, magnesium, phosphorus, potassium, sodium, zinc (40). Spirulina is used in biotechnology to produce and accumulate specific bioactive compounds and nutrients. In this way, a new type of dietary supplement was developed that can serve as a rich source of trace elements (41). In the study, hamsters on a high-fat diet received either SES or unfortified spirulina (both at a dose of 57 mg/kg body weight) daily. This corresponded to a daily dose of 0.57 mg of silicon/kg body weight. The high-fat diet induced dyslipidemia, insulin resistance, oxidative stress, and vascular dysfunction. Compared with the high-fat diet group, SES attenuated the increase in lipemia and prevented insulin resistance (*p* = 0.001), which was not observed in the unsupplemented spirulina group. Another significant effect seen only in the SES group was the reduction of inflammation by lowering the levels of TNF-α (*p* = 0.0006) and interleukin-6 (*p* = 0.0112), reducing the number of polymorphonuclear cells and preventing the increase in the activity of nuclear factor kB (*p* = 0.0259). SES more significantly than spirulina alone corrected the increased plasma levels of the monocyte chemotaxis protein MCP-1 by the high-fat diet (*p* = 0.0380). Furthermore, only SES supplementation prevented the attenuation of aortic vascular and endothelial responses induced by a high-fat diet, as assessed by the contractile response to the phenylephrine agonist and the relaxation response to acetylcholine, respectively. However, both SES and spirulina itself similarly protected against oxidative stress by reducing the activity of nicotinamide adenine dinucleotide phosphate oxidase in the heart and liver and maintaining the activity of the antioxidant enzymes superoxide dismutase and glutathione peroxidase.

### 3.7 Silicone effects on redox status

Observed by Gonzalez-Munoz et al. (38) the effect of silicon on the redox system in mice receiving silicic acid expressed by inhibiting the aluminum-induced decrease in mRNA expression of endogenous antioxidant enzymes, normalizing the reduced levels of the antioxidant enzymes catalase and superoxide dismutase in brain tissue homogenates and reducing the level of free oxygen radicals (TBARS, thiobarbituric acid reactive substances) was confirmed in further studies. Other researchers similarly found that the redox state in rats with nonalcoholic steatohepatitis improved with silicon administration. Increased gene expression of liver antioxidant enzymes and decreased levels of glutathione persulfide were observed (42).

In 2018, Gangapatnam et al. (22) demonstrated regression of atherosclerosis in experimental animals. The research was carried out in the following five groups: group I - normal diet, control group; group II - high-fat diet; group III - statins as a positive control; group IV – high-fat diet + silicon treatment at a concentration of 10 mg/ml; group V - high-fat diet + silicon treatment at a concentration of 20 mg/ml. Levels of physical, biochemical and serum marker enzymes were assessed. A significant difference was found between rabbits on the high-fat diet and the other treatment and control groups. Histopathological examinations were then performed and the levels of antioxidant enzymes such as catalase, glutathione peroxidase and superoxide dismutase in the liver tissue were assessed. The results showed that there were significant differences, with silicon at a concentration of 20 mg/ml showing regression of atherosclerosis in rabbits induced with a high-fat diet compared to a concentration of 10 mg/ml.

The beneficial effect of silicon on the antioxidant system may also be strengthened through its protective effect on the structure of proteoglycans, which themselves have an antioxidant properties (43). The polysaccharides extracts of four of the most widely known mushrooms often used in medicinal applications named Ganoderma applanatum, Ganoderma lucidum, Lentinus edodes and Trametes versicolo showed an increase in the antioxidant activities and inhibition of lipid peroxidation with increasing concentrations (44). The heteropolysaccharides supplemented in medium and high doses to rats with MetS significantly reduced the level of prooxidants (superoxide anion radical O_2_^−^, hydrogen peroxide H_2_O_2_, TBARS; *p* < 0.05) and increased antioxidants activity (superoxide dismutase, catalase, reduced glutathione; *p* < 0.05) compared to untreated MetS animals (20). Mushroom polysaccharides have been shown to mitigate oxidative stress also in type 2 diabetes mellitus by the decreased lipid peroxidation and the increased activity of superoxide dismutase in the plasma, and by the elevated glutathione levels in the plasma and erythrocytes (45).

It is also worth mentioning that the role of silicon in enhancing the antioxidant capacity, including the activity of antioxidant enzymes, has been confirmed for plants exposed to abiotic stress (46).

The few studies on the effect of silicon on the redox system in humans have been conducted in patients with rheumatoid arthritis (RA) (10). Increased dietary silicon intake and higher serum levels have been observed in patients with RA. Under conditions of intense oxidative stress in patients with RA, a correlation was found between silicon in the diet and serum and serum markers of the redox state, which was expressed by lower levels of hydrogen peroxide and lipid peroxide measured in the total oxidant status (TOS) test and was more pronounced in women with an increased content of silicon in their diet. Moreover, the ratio of oxidant and antioxidant status decreased as silicon intake increased in these individuals, indicating a role for Si in managing RA-related oxidant overproduction. In RA patients, these correlations were found only in people without factors affecting the redox state, i.e., lower albumin concentration and cigarette smoking. The observed relationship could be the result of the composition of foods providing silicon, which are mainly of plant origin and contain a wealth of antioxidants with proven effectiveness in neutralizing reactive oxygen species. However, in the light of the studies on animal models and cell lines discussed above showing that silicon has the ability to remove hydrogen peroxide or other reactive oxygen species (36, 37, 47), its protective effect against peroxide toxicity seems likely also in humans. A study of RA patients also demonstrated a negative correlation between silicon and IL-6 in the RA group, indicating its role in preventing bone, cartilage and joint destruction in RA. In patients with RA, a negative correlation was also found between silicon in plasma and the number of swollen joints. Moreover, there was a non-significant positive trend in the duration of the disease among non-smokers. These findings may support a role for silicon in the disease response in RA patients. The role of silicon in the inflammatory process in humans is also suggested by observations of patients with chronic osteoarthritis (48), which showed significantly higher plasma silicon concentrations in the study group compared to the control group of healthy people. These differences were particularly pronounced in men. In males, symptoms of osteoarthritis decreased and were accompanied by significant reductions of cartilage degradation markers. No such effect was observed in women, most of whom were postmenopausal in this study. The influence of hormones on silicon metabolism is still an unexplained issue.

### 3.8 Silicon and diabetes mellitus

In animal model, silicon demonstrated novel antidiabetic effects by lowering blood glucose levels and improving tolerance to insulin, leptin and adiponectin (49). Strengthening the structure of proteoglycans with silicon may have additional beneficial anti-atherosclerotic effect, as heteropolysaccharides also has been shown to regulate glucose metabolism (20, 45, 50).

**Figure 1:**
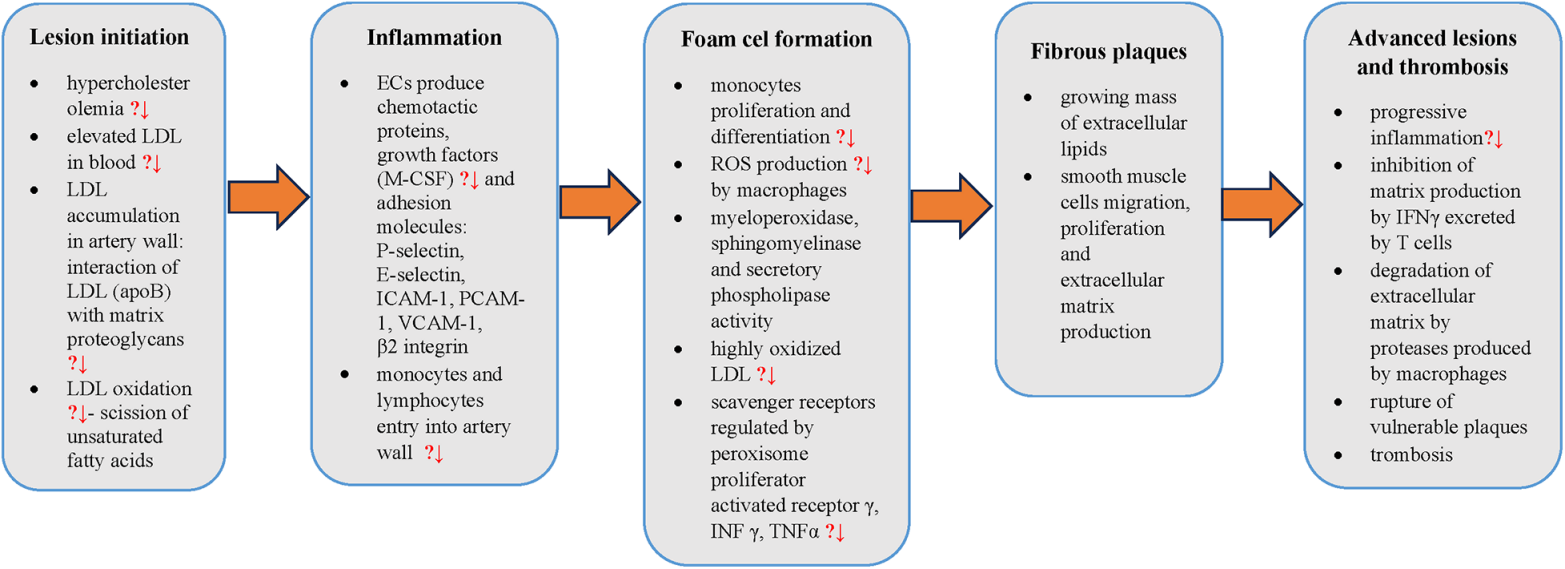
Steps in atherosclerosis pathogenesis with points to potential silicon preventive action (?↓). LDL, low density lipoprotein cholesterol; apoB, apolipoprotein B; ECs, endothelial cells; M-CSF, macrophage colony-stimulating factor; ICAM-1, intercellular adhesion molecule 1; PCAM-1, platelet cell adhesion molecule 1; VCAM-1, vascular cell adhesion molecule 1; ROS, reactive oxygen species; INFγ, interferon γ; TNFα, tumor necrosis factor-α,?↓ - potential decrease.

The potential beneficial effects of silicon in atherosclerosis pathogenesis and possible sites of action have been summarized in Figure 1.

## 4 The relationship of silicon with diseases of the connective tissue, osteoarticular and nervous systems

### 4.1 Silicon and the function of connective tissue and the osteoarticular system

Silicon plays an important role in the formation and maintenance of connective tissue, including collagen and elastin. This element occurs in various organs, including: skin, bones, hair and nails, arteries. The role of silicon in bone formation was already demonstrated in the 1970s in animal studies (51, 52). Soon, *in vitro* studies (17) showed that Si binds to glycosaminoglycan macromolecules and plays a role in creating cross-links between collagen and proteoglycans, which results in the stabilization of extracellular bone matrix molecules and the prevention of their enzymatic degradation. Studies on cell cultures (53) also showed that silicon is necessary for the formation of glycosaminoglycans; on day 8 of observation, the increase in hexosamine content in silicone-supplemented bones was almost 200% greater than in bones with low silicon content. In animal studies (54), the effect of silicon on bone formation was found to be essentially independent of the effects of vitamin D. All chicks on the silicon-deficient diet, regardless of dietary vitamin D levels, had severe abnormalities in skull architecture (the bone matrix was completely devoid of the normal striated trabecular pattern, less calcified, and more transparent); furthermore, silicon-deficient skulls showed significantly less collagen at each level of vitamin D. The above studies suggested that the main effect of silicon on bone involves the formation of both glycosaminoglycans and collagen in the connective tissue matrix and is independent of vitamin D.

There are many reports in the scientific literature regarding the active role of silicon in bone mineralization and the prevention of osteoporosis (4, 55, 56). The first large population-based study on 1,251 men and 1,596 pre- and post-menopausal women from the Framingham Offspring cohort (age 30–87 years) was conducted by Jugdaohsingh et al. (4), in which they showed a positive correlation between silicon intake and adjusted bone mineral density (BMD) marked in four hip sites in men and women of premenopausal age. No such correlation was found in the group of postmenopausal women or in any of the groups with respect to the BMD of the lumbar spine. Categorical analysis by Si intake or energy-adjusted Si intake confirmed these observations and demonstrated extensive differences in BMD (up to 10%) between the highest (>40 mg Si/day) and lowest (<14 mg Si/day) silicon intake quintiles.

Similar results were obtained by Macdonald et al. (56) - a study on a cohort of 3,198 women aged 50–62 years showed a significant relationship between dietary silicon intake and hip bone mineral density in women currently using hormone replacement therapy (HRT) and for premenopausal women, but not for women with estrogen deficiency (postmenopausal women not taking HRT). This may suggest that the correct level of estrogen, especially estradiol, is necessary for the proper functioning of silicon in the skeletal system.

Also a 2-year follow up pilot study (57) showed that beer consumption did not cause a difference in bone mineral density in early postmenopausal women compared to the control group not consuming beer, although bone formation markers (bone alkaline phosphatase and type I collagen N-propeptide) increased in relation to the control group.

The beneficial effect of silicon on the skeletal system may be due to the effect of orthosilicic acid on osteoblasts. An *in vitro* study (13) demonstrated that orthosilicic acid at physiological concentrations stimulates the synthesis of type 1 collagen in human osteoblast-like cells and fibroblasts of skin and also strengthens osteoblast differentiation. Expression of the type 1 collagen gene was not changed by the orthosilicic acid, but the results suggested that the activity of prolyl hydroxylase, a key enzyme for the synthesis of type 1 collagen may be modulated by this silicon compound. The mechanism of the interaction between soluble silicon and prolyl hydroxylase is unclear.

### 4.2 Silicon and the nervous system

**Figure 2:**
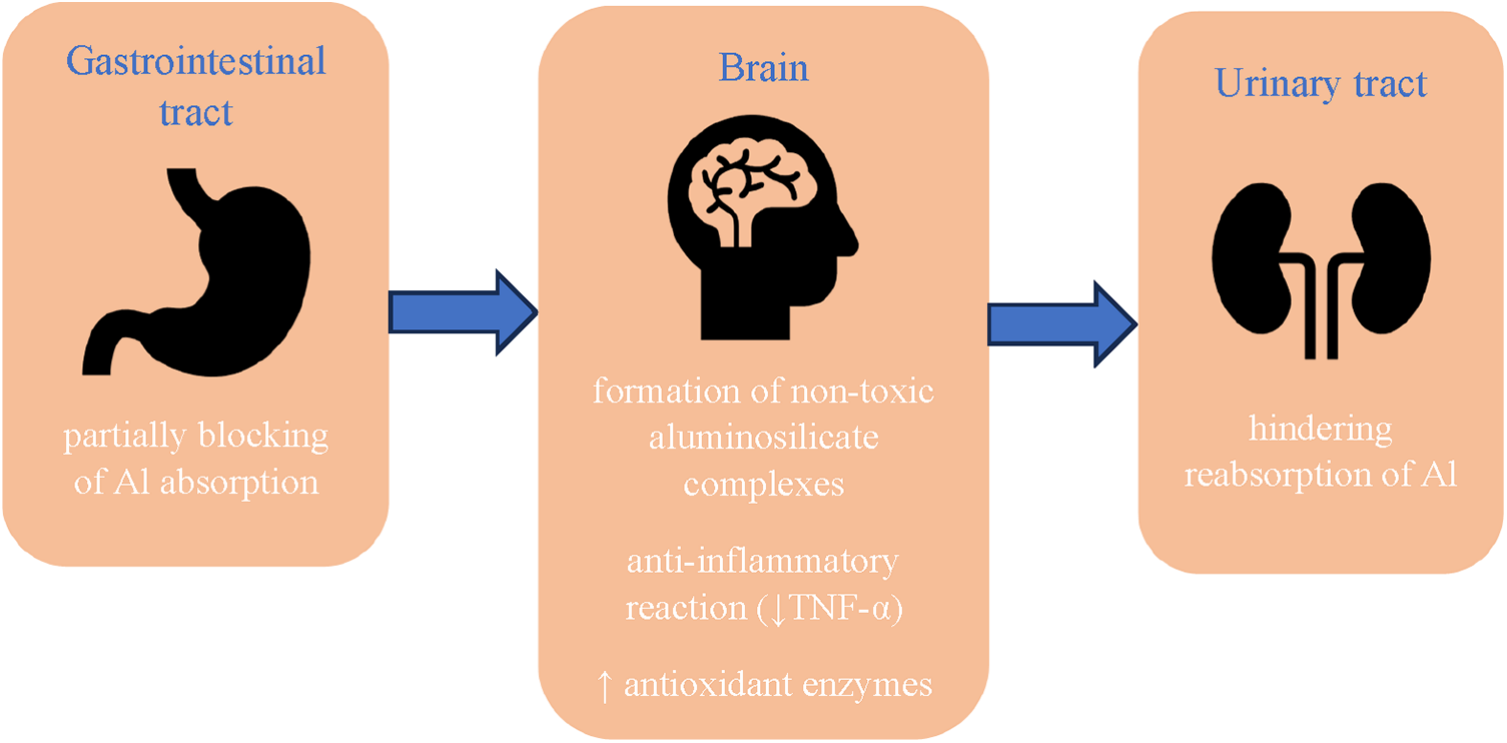
Protective effect of silicon against the development of neurodegenerative diseases. Al, aluminum; TNF-α, tumor necrosis factor-α.

Silicon may have a protective effect against the development of neurodegenerative diseases. In recent years, many studies have appeared which indicate that aluminum is one of the risk factors for Alzheimer's disease. Experimental studies in rats and mice have shown that aluminum accumulates in the cerebral cortex, hippocampus and cerebellum (58), subsequently promoting the aggregation of highly phosphorylated proteins such as tau (1). According to Kawahara (59), this metal induces neuronal apoptosis both *in vivo* and *in vitro*. The mechanism of the toxic effect of aluminum on the brain has not been finally elucidated, but the following factors are considered: cross-linking of hyperphosphorylated proteins, leading to the formation of tau protein (60); promoting the expression of amyloid precursor protein (APP) and increasing the level of fragments β-40 and β-42 in the brain, which may lead to the formation of neurofibrillary tangles characteristic of the disease (61); increase in oxidative stress and inflammatory reaction due to decreased activity of antioxidant enzymes (catalase, superoxide dismutase, glutathione peroxidase) (62); damage to the cholinergic system (63). Silicon and silicic acid reduce the bioavailability of aluminum by partially blocking its absorption in the gastrointestinal tract (64, 65) and hindering its reabsorption (66) (Figure 2). A controlled clinical study of Alzheimer's disease showed that drinking up to 1 liter of silicon-rich mineral water daily for 12 weeks increased urinary aluminum excretion in both patients and controls, without any concomitant effect on urinary excretion of essential metals, iron and copper (67).

The effectiveness of silicon in the prevention of Alzheimer's disease can also be attributed to its interaction with aluminum through the formation of non-toxic aluminosilicate complexes, which reduce the availability of free aluminum not only during absorption in the gastrointestinal tract, but also in the cells. A number of biological sites have been identified where silicon and aluminum are colocalized. The enlarged Si content (3–50 times) has been revealed in the cores and rims of senile plaques in the cerebral cortex of patients suffering from senile dementia/Alzheimer's disease type (68). Measurements in the central areas of senile plaques have shown that silicon and aluminum are present in the form of aluminosilicates (69), what possibly can be a way to curtailing of the toxicity of aluminum (38). Senile plaque structures have also been observed in mentally healthy elderly patients and the use of dietary silicon supplements has been suggested as a preventive measure for Alzheimer's disease (9).

Other authors (5) have examined correlation between exposure to aluminum or silica from drinking water and the risk of dementia, cognitive decline and Alzheimer's disease among older adults. They analyzed 1,925 people who were followed for 15 years (1988–2003). Using random models, the authors demonstrated that cognitive decline over time was larger in individuals with greater daily intake of aluminum from drinking water (≥0.1 mg/day, *p* = 0.005) or greater geographic exposure to this element. On the contrary, increasing silicon intake by 10 mg/day was related to reduced risk of dementia (adjusted relative risk = 0.89, *p* = 0.036) and especially Alzheimer's disease.

Single *in vitro* study (37) reports a neuroprotective action of low silicon concentrations on human SH SY5Y neuroblastoma cell lines by inducing an anti-apoptotic effect and anti-inflammatory reaction as a result of diminishing the level of tumor necrosis factor TNF-α. Also animal studies (38) showed that beer and silicic acid inhibit the aluminum-induced decrease in mRNA expression of endogenous antioxidant enzymes and normalize the expression of TNF-α mRNA. Beer also significantly prevented the accumulation of lipid damage that resulted from aluminum consumption. This finding is important because increased levels of oxidative stress and lipid peroxidation products in brain tissue are major factors contributing to the development of neurodegenerative diseases. In subgroups of animals receiving silicic acid and beer, decreased levels of the antioxidant enzymes catalase and superoxide dismutase in brain tissue homogenates normalized, while levels of thiobarbituric acid reactive substances (TBARS) decreased.

## 5 Conclusions

The role of silicon in the pathogenesis of atherosclerosis and its complications remains controversial (60). Most of the works indicating the involvement of this element in atherogenesis processes come from animal studies. Currently, the number of studies in humans regarding the relationship between silicon and established risk factors for atherosclerosis, as well as the possible impact on individual cells involved in the process of initiation, progression and destabilization of atherosclerotic plaque is very limited. However, taking into account the above-mentioned results of animal studies indicating beneficial changes in the lipid profile after the use of silicon, adequate dietary intake of the element may contribute to inhibiting the progression of atherosclerosis. Moreover, the beneficial immunomodulatory effect of the element demonstrated in an animal model, and in particular the observation of a decrease in the number of monocytes accompanying a higher intake of silicon, may have a significant impact on the course of the disease caused by chronic inflammation. The role of Si is also supported by the relationship observed in patients with rheumatoid arthritis with both the function of the redox system and the value/titer of inflammatory markers. Due to the existing research conducted mainly on cell lines or animal models and the small number of studies in humans, the importance of silicon in preventing atherosclerosis and other age-related diseases requires further in-depth research.
